# Metastasis-on-a-chip mimicking the progression of kidney cancer in the liver for predicting treatment efficacy

**DOI:** 10.7150/thno.38736

**Published:** 2020-01-01

**Authors:** Yimin Wang, Di Wu, Guohua Wu, Jianguo Wu, Siming Lu, James Lo, Yong He, Chao Zhao, Xin Zhao, Hongbo Zhang, ShuQi Wang

**Affiliations:** 1 State Key Laboratory for Diagnosis and Treatment of Infectious Diseases, National Clinical Research Center for Infectious Diseases, Collaborative Innovation Center for Diagnosis and Treatment of Infectious Diseases, The First Affiliated Hospital, College of Medicine, Zhejiang University, Hangzhou, Zhejiang Province, 310003, China.; 2 Institute for Translational Medicine, Zhejiang University, Hangzhou, Zhejiang Province, 310029, China.; 3 Department of Bioengineering, University of California Berkeley, Berkeley, CA, 94720, United States of America.; 4 State Key Laboratory of Fluid Power and Mechatronic Systems, Key Laboratory of 3D Printing Process and Equipment of Zhejiang Province College of Mechanical Engineering, Zhejiang University, Hangzhou, Zhejiang Province, 310029, China.; 5 Wellcome Trust-Medical Research Council Cambridge Stem Cell Institute and Department of Clinical Neurosciences, University of Cambridge, Cambridge CB2 0AH, United Kingdom.; 6 Department of Biomedical Engineering, The Hong Kong Polytechnic University, Hong Kong SAR, China.; 7 Department of Pharmaceutical Science, Åbo Akademic University, FI-20520, Turku, Finland.

**Keywords:** Metastasis-on-a-chip, Tumor progression, Chemotherapy, Drug delivery.

## Abstract

Metastasis is one of the most important factors that lead to poor prognosis in cancer patients, and effective suppression of the growth of primary cancer cells in a metastatic site is paramount in averting cancer progression. However, there is a lack of biomimetic three-dimensional (3D) *in vitro* models that can closely mimic the continuous growth of metastatic cancer cells in an organ-specific extracellular microenvironment (ECM) for assessing effective therapeutic strategies.

**Methods:** In this metastatic tumor progression model, kidney cancer cells (Caki-1) and hepatocytes (*i.e.,* HepLL cells) were co-cultured at an increasing ratio from 1:9 to 9:1 in a decellularized liver matrix (DLM)/gelatin methacryloyl (GelMA)-based biomimetic liver microtissue in a microfluidic device.

**Results:** *Via* this model, we successfully demonstrated a linear anti-cancer relationship between the concentration of anti-cancer drug 5-Fluorouracil (5-FU) and the percentage of Caki-1 cells in the co-culture system (R^2^ = 0.89). Furthermore, the Poly(lactide-co-glycolide) (PLGA)-poly(ethylene glycol) (PEG)-based delivery system showed superior efficacy to free 5-FU in killing Caki-1 cells.

**Conclusions:** In this study, we present a novel 3D metastasis-on-a-chip model mimicking the progression of kidney cancer cells metastasized to the liver for predicting treatment efficacy. Taken together, our study proved that the tumor progression model based on metastasis-on-a-chip with organ-specific ECM would provide a valuable tool for rapidly assessing treatment regimens and developing new chemotherapeutic agents.

## Introduction

Although significant advances have been made to save cancer patients' lives, metastasis is still the leading cause of cancer-related mortality, accounting for approximately 90% of global cancer deaths 1. As reported, the 5-year survival rate of patients with a primary tumor is relatively high, whereas metastasis can considerably reduce the 5-year survival rate to less than 30% 1, 2. Chemotherapy remains the mainstream treatment strategy for metastatic cancer, particularly when the primary tumor cannot be surgically removed 3, 4. Although chemotherapy plays a key part in the plethora of options for the clinical management of metastatic cancer, the success rate of developing effective chemo-compounds is less than 5% due to the limitation of conventional models 5. The selection of highly effective anticancer treatment rather than empirical treatment is much needed for curbing cancer progression in metastatic cancer patients. It is, therefore, essential to identify effective anticancer chemotherapies and optimize drug delivery efficiency for improved clinical outcomes in metastatic cancer patients.

Traditionally, two-dimensional (2D) cell cultures and animals have been used as primary tumor and metastatic cancer drug development models. Although 2D cell culture is relatively easy to perform, it lacks the essential physiopathological cues in the tumor microenvironment and metastatic niche 6, 7. As such, the inadequacies of traditional 2D models lie in their inability to accurately simulate the complex, interact with appropriate physiopathological conditions or predict the *in vivo* effectiveness of anticancer compounds 8-10. On the other hand, animal models for drug testing are labor/time intensive, costly, and most importantly, often yield untranslatable results due to the physiological differences between humans and animal models 11-14. Therefore, the creation of cost-effective, reliable, and pragmatic *in vitro* models that can be used for accurately screening anticancer drug effects as well as overcoming the drawbacks of conventional models is of great importance for improving the current clinical management of primary tumor and metastatic cancer 15-18.

In recent years, organ-on-chip platforms, due to their 3D nature and cost-effectiveness, have been developed to model the metastatic cascade within conditioned microenvironments 19-23. Metastasis-on-a-chip models have been used to study the metastatic cascade and offer a feasible platform for drug testing 24-26. For instance, a metastasis-on-a-chip was constructed to mimic the migration of metastatic tumors from the intestine to the liver and to allow real-time tracking of cell movement and behavior 27. However, this study only used hyaluronic acid hydrogel without considering organ-specific ECM in the migration model. In another study, normal breast cells are co-cultured with breast cancer cells to simulate cancer models at mild, moderate, and severe stages, in which cell density is found to be highly correlated with the incidence of metastasis 28. However, this study used a 2D rather than a 3D model to investigate cancer migration and drug screening. Therefore, the progression of post-metastasis tumor within an organ-specific ECM has not been studied.

In this article, we present a new metastasis-on-a-chip model incorporated with organ-specific ECM. This model can mimic the progression of kidney cancer cells in the liver to predict the therapeutic effects and evaluate dosage responses of anticancer drugs in a physiologically relevant liver microenvironment. We cultured kidney cancer cells (Caki-1) in a DLM/GelMA-based 3D biomimetic liver microtissue via continuous perfusion. Within this model, we co-cultured the Caki-1 and HepLL cells in increasing ratios from 1:9 to 9:1 to investigate the progression of metastatic kidney cancer cells in the liver. We observed that there was a linear anticancer relationship between the concentration of 5-Fluorouracil (5-FU) and the percentage of Caki-1 cells in the metastatic tumor progression model, and that the 5-FU-loaded PLGA-PEG nanoparticles (NPs) showed a stronger killing efficacy than free 5-FU. Our findings demonstrate that the tumor progression model can be used to establish 3D metastatic cancer *in vitro* models and to rapidly assess anti-cancer efficiency and optimize dosage regimes.

## Methods

### Decellularization and characterization of DLM

The decellularized scaffold of liver was prepared according to the method previously described 29. The use of Sprague-Dawley rats and their livers was approved by the Zhejiang University Experimental Animal Welfare Ethics Committee (ZJU20170787). For scanning electron microscope (SEM) imaging, both the native livers and the DLM were frozen and maintained at -20 °C for 12 hours before lyophilization for 24 hours. The native livers and DLM were observed and analyzed with the aid of a SEM (Hitachi, Tokyo, Japan).

The H&E and immunofluorescence staining were performed after sectioning native liver tissues and DLM. The sectioned samples (n = 3) were then stained with a first rabbit polyclonal antibody against collagen type I, collagen type IV, fibronectin or laminin (Abcam, Cambridge, UK). Then, sectioned samples were stained with a second goat anti-rabbit antibody labeled with Alexa Fluor® 594 (Abcam, Cambridge, UK). Lastly, the nuclear DNA was detected using with 4', 6-diamidino-2-phenylindole dihydrochloride (DAPI) (Sigma-Aldrich, St. Louis, US). A fluorescence microscope (AXIO Observer A1, Zeiss, Oberkochen, Germany) was used to image the fluorescence-stained samples.

### Measurement of Young's modulus

Young's modulus was measured as an indicative of stiffness in order to characterize 100% HepLL, H/C=9/1, H/C=7/3, H/C=5/5, H/C=3/7, H/C=1/9, and 100% Caki-1 cells-laden hydrogels with a mass ratio of 2:3 of DLM/GelMA, in which the concentration of GelMA was 0.1 g/mL. The hydrogels were molded into a shape of cylinder with dimensions of 9 mm in height and 10 mm in diameter. The Young's modulus of the hydrogels was measured at a 1 mm/min rate under a mechanical tester (EFL-MT5600, Suzhou Intelligent Manufacturing Research Institute, Suzhou, China).

### Transduction of HepLL and Caki-1 cells

A total number of 50,000 cells of HepLL and Caki-1 (a representative cell line derived from human kidney cancer, which has been commonly used to establish xenograft mouse renal cell carcinoma(RCC) models by subcapsular implantation 30, 31) were added to a 24-well plate, and 500 μL of DMEM (Gibco, Melbourne, Australia) was added to each well. After 24 hours, the pLenti-CMV-EGFP-3FLAG-PGK-Puro and pLenti-CMV-mCherry-3FLAG-PGK-Puro lentivirus (ObiO Technology, Shanghai, China) were added to HepLL and Caki-1 cells, respectively. After the virus was introduced to the cells for 12 hours, the medium was replaced. After 72 hours of virus introduction, the cells were observed under a microscope to confirm that the lentivirus were successfully transduced into the target cells. After a 3-day puromycin (Sangon Biotech, Shanghai, China) treatment, the uninfected cells were removed.

### Preparation of microfluidic device for metastatic tumor progression model

A microfluidic device, on which a metastatic tumor progression model was established, was assembled using poly (methyl methacrylate) (PMMA) for structural and spatial components. Poly (dimethylsiloxane) (PDMS), a hydrophobic elastomer with excellent gas permeability, was incorporated to facilitate gas exchange to the cells. The CorelDraw® system was used to design each layer of PMMA and a laser cutting machine (Universal Laser System, Scottsdale, US) was used to prepare the device containing an inlet and outlet as well as 7 microwells for 3D cell culture. For preparing the PDMS component, the precursor of PDMS and coagulant were mixed at a ratio of 10:1. After degassing for 30 minutes, the mixture was then cured in a mold at 80 °C for 1 hour. A PDMS sheet was obtained after peeling. A PET membrane with a pore size of 10 μm (GE Healthcare Life Science, Shanghai, China) was sliced to creating the required form-fitting shapes to be incorporated directly above the microwells. The 3M Scotch-Weld adhesive was used in conjunction with screws to assemble the microfluidic device with multiple layers of PMMA and PDMS.

### Long-term cell culture in metastatic tumor progression model

Prior to cell seeding, the assembled devices were initially sterilized with 75% ethanol over the course of 24 hours and then sterilized with 2.5 μg/mL amphotericin integrated into 2% penicillin-streptomycin (Sangon Biotech, Shanghai, China) over a course of 2 hours.

DMEM containing 10% fetal bovine serum (FBS) (Gibco, Melbourne, Australia) was subsequently used to suspend the HepLL and Caki-1 cells at varying ratios (100% HepLL, 9: 1 H/C, 7: 3 H/C, 5: 5 H/C, 3: 7 H/C, 1: 9 H/C and 100% Caki-1 cells) with a final concentration of 1.0 × 10^7^ cells per/mL. The cell suspension underwent 2 minutes of centrifugation at 1,000 rpm and the same volume of 2:3 DLM/GelMA solution was used to replace the medium. The mixture was then transferred to the microfluidic device before exposure to ultraviolet (UV)-light for 30 seconds at a wavelength of 365 nm for solidification. DMEM was continuously flowed into the upper chamber of the microfluidic devices at a rate of 2 μL/h. For media perfusion and fluidic connection, silicone tubing (0.5 mm ID × 2.0 mm OD, Longer Pump, Baoding, China) was used.

On days 1, 7, and 14, Live/Dead assays (Dojindo, Kumamoto, Japan) were used to evaluate the cell viability as described by the manufacturer's instructions. A Nikon A1 confocal fluorescence microscope (Nikon, Tokyo, Japan) was used to analyze the stained cells, and all images produced were analyzed with the ImageJ software. The percentage of live cells among all the seeded cells was used to evaluate cell viability. Furthermore, the transduced HepLL and Caki-1 cells were co-cultured for 7 days and then observed under a Nikon A1 confocal fluorescence microscope to confirm the success of co-culture.

### Measurement of urea and albumin (ALB) secreted by liver cells

The secretion of urea and ALB was quantified to assess hepatocyte function. The supernatant was collected from the co-culture on days 1, 7, and 14 with the DMEM media being replaced every 7 days. ELISA assay kits (Luding Biotechnology, Shanghai, China) were employed to quantify the level of secreted urea. ELISA assay kits (Gene beauty Technology, Wuhan, China) were also employed to quantify the level of secreted ALB, according to the manufacturers' instructions. Measurements were taken with a SpectraMax M5 (Molecular Devices, San Jose, US) multifunctional microplate reader.

### Efficacy measurement of 5-FU on metastatic tumor progression model

Using the method described above, the HepLL and Caki-1 cells were homogenously combined into 2:3 DLM/GelMA; it was then injected into the assembled devices to assess drug responses. The devices were incubated in 5% CO_2_ over the course of 48 hours at 37 °C. All drug toxicity assessments were conducted through continuously exposing the HepLL and Caki-1 cells to media containing 0, 20.0, 40.0, and 60.0 μg/ mL of 5-FU (MedChem Express, Monmouth Junction, NJ, USA) at a rate of 2 μL/h for 24 hours. Cell viability was determined using the CCK-8 kit (MedChem Express, Monmouth Junction, NJ, USA) with the absorbance being measured at a wavelength of 450 nm.

### Preparation of 5-FU-loaded PLGA-PEG NPs

5-FU-loaded PLGA-PEG NPs were produced through the nanoprecipitation method 32. Briefly, 10 mg of 5-FU and 15 mg of PLGA (20 kDa) -PEG (5 kDa) polymer (Sigma-Aldrich, St. Louis, MO, USA) were first added into 1 mL of DMSO, as an organic phase, for thorough dissolving. Then, 1 mL of the organic mixture was added dropwisely into 10 mL of Milli-Q water at a rate of 0.2 mL/min via a syringe pump with constant magnetic stirring for 30 minutes. The obtained suspension was then passed through a 0.45 μm pore size filter once and a 0.22 μm pore size filter twice to remove drug aggregates and to obtain NPs of the desired size and low polydispersity. The NP solution was then dialyzed via a dialysis membrane with a molecular weight cutoff of 12-14 kDa overnight to remove the remaining organic solvents from the solution. The NP solution was then collected and stored at 4 °C.

### Characterization of 5-FU-loaded PLGA-PEG NPs

The size distribution of 5-FU-loaded PLGA-PEG NPs was measured using a dynamic light scattering (DLS) instrument (Zetasizer Nano S-90, Malvern, Worcestershire, UK) at ambient temperature. The angle of detection was set to 90°, and the wavelength of the helium/neon laser was 633 nm. The size of the NPs was determined according to the Stokes-Einstein equation.

5-FU-loaded PLGA-PEG NPs were imaged with the aid of transmission electron microscopy (TEM) (Hitachi, Tokyo, Japan). A 2% (v/v) aqueous phosphotungstic acid solution was used to negatively stain the NP suspension for 3 minutes. A drop (approximately 2 μL) of the stained NP suspension was added to a formvar-carbon coated copper electron microscopy grid. After drying, the stained samples were then imaged with TEM at 50,000× magnification.

The encapsulation efficiency (EE) of 5-FU into NPs was determined using the following protocol. The 5-FU-loaded PLGA-PEG NP suspension was centrifuged (8,000 rpm, 10 min) briefly using an Amicon Ultra-0.5 centrifugal filter (10K cutoff), followed by resuspension of the NPs containing encapsulated 5-FU in ethanol (2 mL). A SpectraMax M5 multifunctional microplate reader was used to measure the concentration of the 5-FU in the mixture by obtaining the UV absorbance level of the above solution at 266 nm to calculate the drug mass in the PLGA-PEG. The EE was expressed as the percentage of encapsulated amount of drug (E_drug_) in the total amount of drug used (T_drug_) as follows 33:


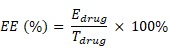


### Delivery of Coumarin 6 by PLGA-PEG NPs

Coumarin 6 (C6) (Sigma-Aldrich, St. Louis, MO, USA) -loaded PLGA-PEG NPs were primed as mentioned but by substituting 5-FU with C6 within the organic phase. HepLL and Caki-1 cells were placed into a 96-microwell plate with a cell density of 6 × 10^4^ cells/mL. After incubating for 24 hours, the medium was purged and refreshed with either 20 μL of the prepared C6-loaded PLGA-PEG NP solution or 20 μL of C6 dissolved in water at a concentration corresponding to the C6 within the NP solution (determined through UV absorbance as described above). Cells were then incubated for an additional 24 hours. Following incubation, cells were stained with DAPI as instructed by the manufacturer and imaged using a fluorescence microscope (Zeiss, Oberkochen, Germany) at a wavelength of 405 nm for DAPI staining and a wavelength of 505 nm for C6 imaging.

### Delivery of 5-FU by PLGA-PEG NPs in metastatic tumor progression model

The HepLL and Caki-1 cells loaded into 2:3 DLM/GelMA were flowed into the device. After UV-crosslinking, cells were incubated with 5% CO_2_ over the course of 48 hours at 37 °C. The metastatic tumor progression model was continuously exposed to media encompassing 30.0 μg/mL of 5-FU or 5-FU-loaded PLGA-PEG NPs for 24 hours. Finally, the cell viability within the hydrogels was analyzed using a Live/Dead kit and a Nikon A1 confocal fluorescence microscope.

### Statistical analysis

Statistical analysis was performed using the one-way of variance (ANOVA) approach among the obtained data. The significance of the data was determined by a p value that was smaller than 0.05.

## Results

### Characterization of biomimetic liver microenvironment

**Figure 2:**
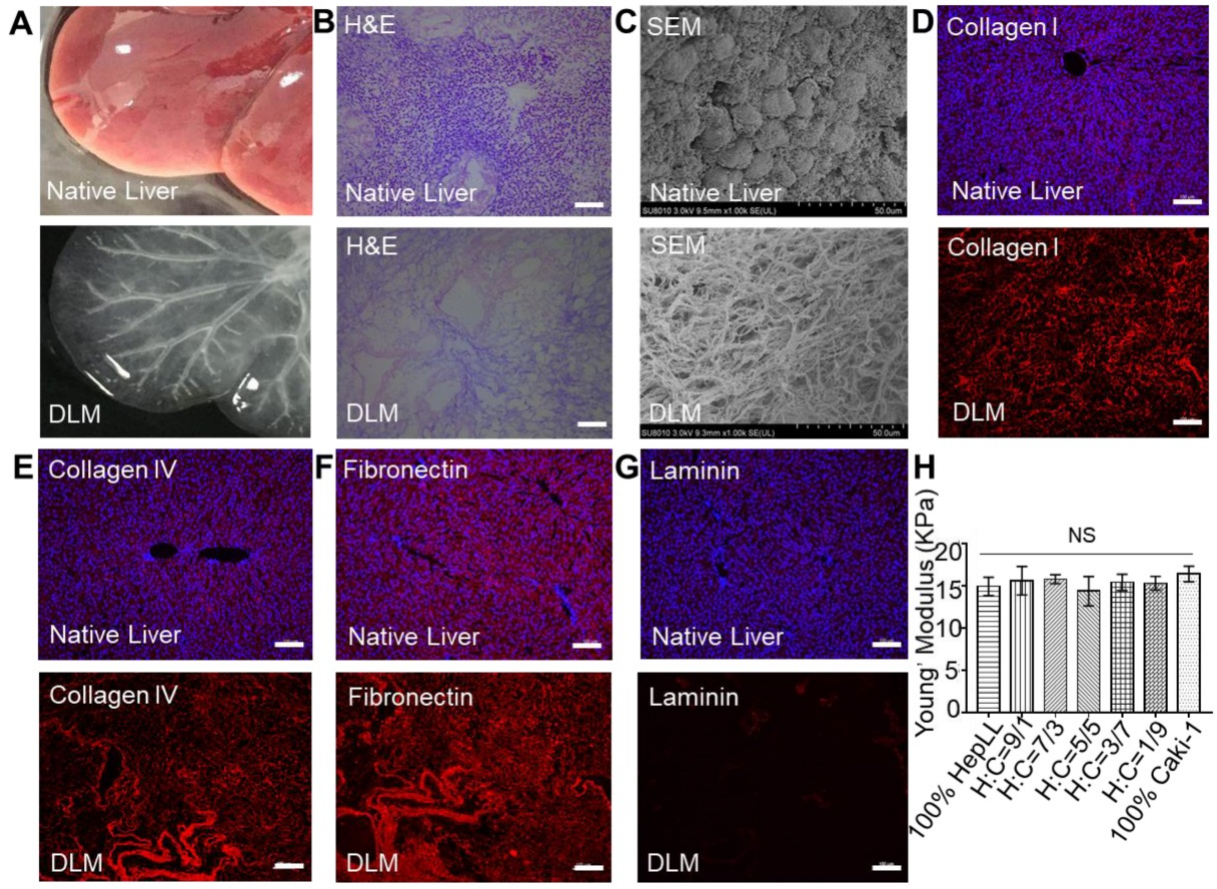
Characterization of the native liver, DLM, and Young's modulus of DLM/GelMA hydrogel. (A) Gross images of a rat liver and DLM. (B) H&E staining of a native liver before and after decellularization. (C) SEM characterization of a native before and after decellularization. (D-G) Immuno-characterization of collagen I (red), collagen IV (red), fibronectin (red), and laminin (red) in the native liver and DLM. Nuclei of the cells were counterstained with DAPI (blue). (H) Measurement of Young's modulus of 2:3 DLM/GelMA hydrogel with varying cell ratios. Scale bar: 100 μm (B, D-G). (Avg. ± SD, NS indicates p > 0.05, n = 3).

The decellularized liver scaffold was obtained to create a biomimetic liver-specific ECM through a previously described protocol 29. The rat livers gradually turned clear and transparent over the course of a 9-hour decellularization process (Figure 2A). After H&E staining, evidence of pink eosinophilic staining denoted the presence of collagen, as opposed to the acellular nature being validated by the lack of basophilic hematoxylin staining (Figure 2B). SEM images also showed the acellular, mesh-like morphology of the decellularized matrices, while visible cells were observed in images of the native liver (Figure 2C). Furthermore, four major ECM proteins (i.e., collagen type I, collagen type IV, fibronectin and laminin) were identified (Figure 2D-G). Moreover, there was no detectable blue staining of nuclei by DAPI, indicating that the decellularization process was successful.

The mechanical properties of the 2:3 DLM/GelMA hydrogels were characterized by gauging their Young's modulus as a biomechanical cue. The results demonstrated that the Young's modulus was 14.93 ± 1.10 kPa, 15.61 ± 1.69 kPa, 15.79 ± 0.52 kPa, 14.37 ± 1.73 kPa, 15.41 ± 0.97 kPa, 15.31 ± 0.81 kPa and 16.41 ± 0.92 kPa for the hydrogels with 100% HepLL, H/C=9/1, H/C=7/3, H/C=5/5, H/C=3/7, H/C=1/9, and 100% Caki-1, respectively (Figure 2H). These data indicated that Young's modulus of the hydrogel did not change significantly for the addition of different proportions of cells. Also, the Young's modulus of the 2:3 DLM/GelMA hydrogel was within the stiffness range of liver cancer (approximately 5 to 60 kPa) 34, 35.

### Long-term 3D cell culture using the metastatic tumor progression model

**Figure 3:**
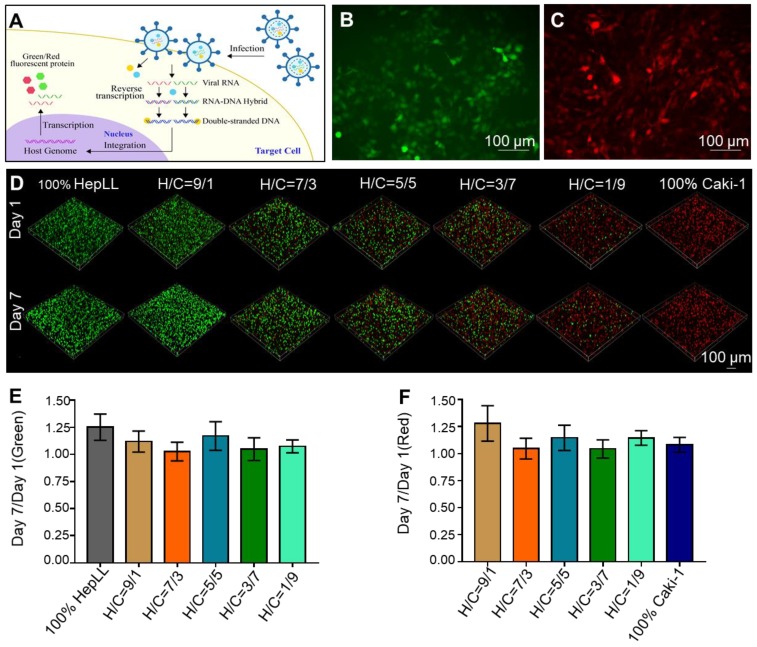
Transduced HepLL and Caki-1 cells co-cultured in the tumor progression model based on metastasis-on-a-chip. (A) Transduction diagram demonstrating the introduction of Green fluorescent protein (GFP) and Red fluorescent protein (RFP). (B) 2D transduction results of HepLL expressing GFP and (C) Caki-1 cells expressing RFP. (D) Confocal images of HepLL and Caki-1 cells co-culture on the tumor progression model based on metastasis-on-a-chip on day 1 and day 7. The ratio of green (E) and red (F) fluorescence expressed on day 7 compared to day 1 (Avg. ± SD, n = 3).

To observe the growth of HepLL and Caki-1 cells, we transduced lentivirus into HepLL (MOI = 30) and Caki-1 (MOI = 20). As a result, HepLL cells expressed green fluorescent protein and Caki-1 cells expressed red fluorescent protein (Figure 3A-C). The ratio of green fluorescence on day 7 to day 1 were 1.25 ± 0.12, 1.12 ± 0.10, 1.03 ± 0.09, 1.17 ± 0.13, 1.05 ± 0.11 and 1.08 ± 0.06 for 100% HepLL, H/C=9/1, H/C=7/3, H/C=5/5, H/C=3/7 and H/C=1/9, respectively. The ratio of red fluorescence on day 7 to day 1 were 1.28 ± 0.16, 1.05 ± 0.10, 1.15 ± 0.12, 1.04 ± 0.08, 1.15 ± 0.07 and 1.08 ± 0.07 for H/C=9/1, H/C=7/3, H/C=5/5, H/C=3/7, H/C=1/9 and 100% Caki-1, respectively. The above results indicated the relatively consistent increase of transduced HepLL and Caki-1 cells in the metastatic tumor progression model (Figure 3D-F).

**Figure 4:**
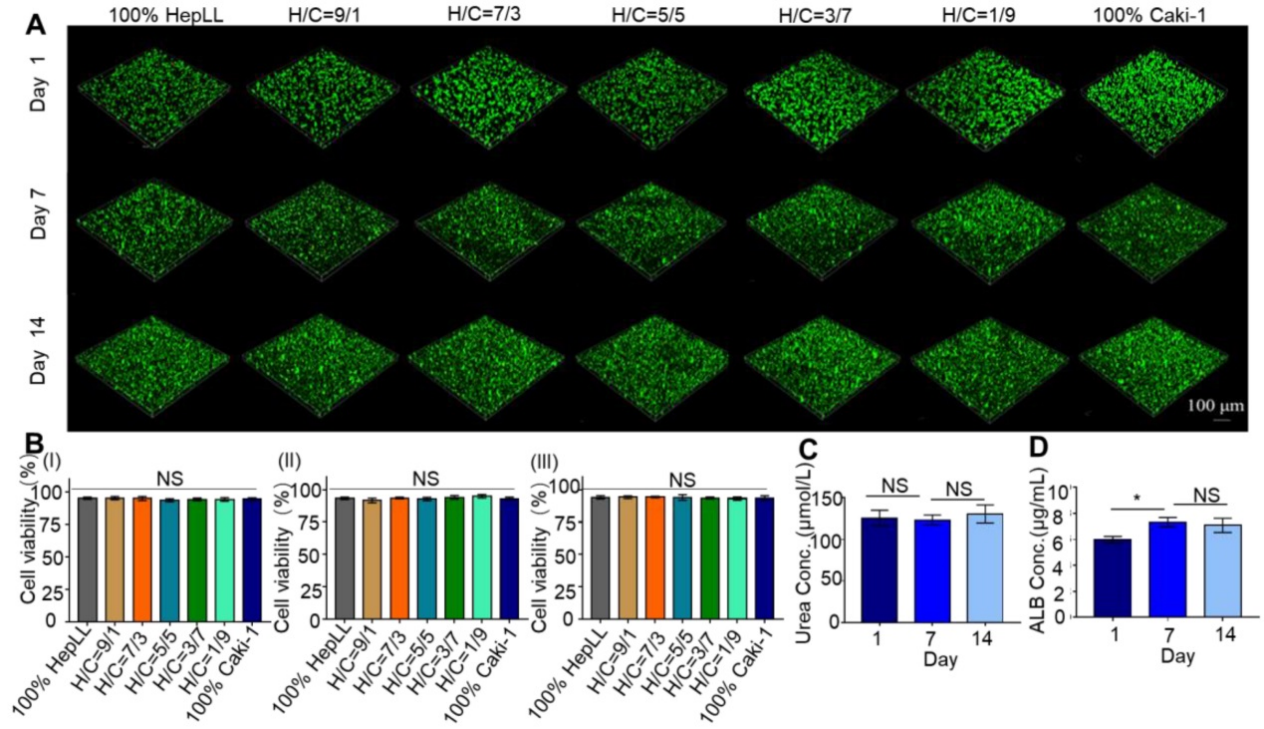
Functional characterization of HepLL and Caki-1-laden DLM/GelMA hydrogels in the tumor progression model based on metastasis-on-a-chip. (A) Live/dead staining of HepLL and Caki-1 within 2.3 DLM/GelMA on days 1, 7, and 14 of cell culture. (B) Analysis of the cell viability on days 1, 7, and 14 (n = 3). (C-D) Measurements of ALB (C) and urea (D) secreted from the tumor progression model based on metastasis-on-a-chip. (Avg. ± SD, * stands for p < 0.05, NS indicates p > 0.05, n = 3).

The cell viability of non-transduced HepLL and Caki-1 cells in DLM/GelMA scaffold was evaluated by live/dead staining. In the metastatic tumor progression model, where the cells were entirely embedded in 3D hydrogels in conjunction with continuous media perfusion, the HepLL and Caki-1 cells were capable of culture for 14 days without a significant reduction in cell viability (Figure 4A). The cell viability values were 94.13 ± 1.20%, 94.23 ± 0.96%, 94.03 ± 0.63%, 93.57 ± 2.16%, 93.16 ± 1.29% and 93.63 ± 1.65% when evaluated on day 14. The viability of total cells within the scaffold at different ratios showed no significant difference (Figure 4B).

### Secretion of urea and ALB by liver cells

The secretion level of urea and ALB from the HepLL cells over a 14-day culture period indicated the occurrence of metabolism and physiologically relevant cellular processes within the liver-specific ECM. Urea was consistently secreted from the non-transduced HepLL cells in the 2:3 DLM/GelMA hydrogels, indicated by the concentrations of 125.74 ± 9.29 μM, 123 ± 6.10 μM and 130.58 ± 10.90 μM on days 1, 7, and 14, respectively (Figure 4C). The urea concentration showed no significant difference over the duration of cell culture. In addition, the secretion of ALB showed a significant increase on day 7, and this level was maintained over the remaining culture period (5.98 ± 0.25 μg/mL, 7.33 ± 0.37 μg/mL, and 7.08 ± 0.55 μg/mL on day 1, 7, and 14 respectively) (Figure 4D). The ALB concentration showed a significant difference on days 1 and 7, but showed no significant difference on days 7 and 14.

### Efficacy of 5-FU on metastatic tumor progression model

**Figure 5:**
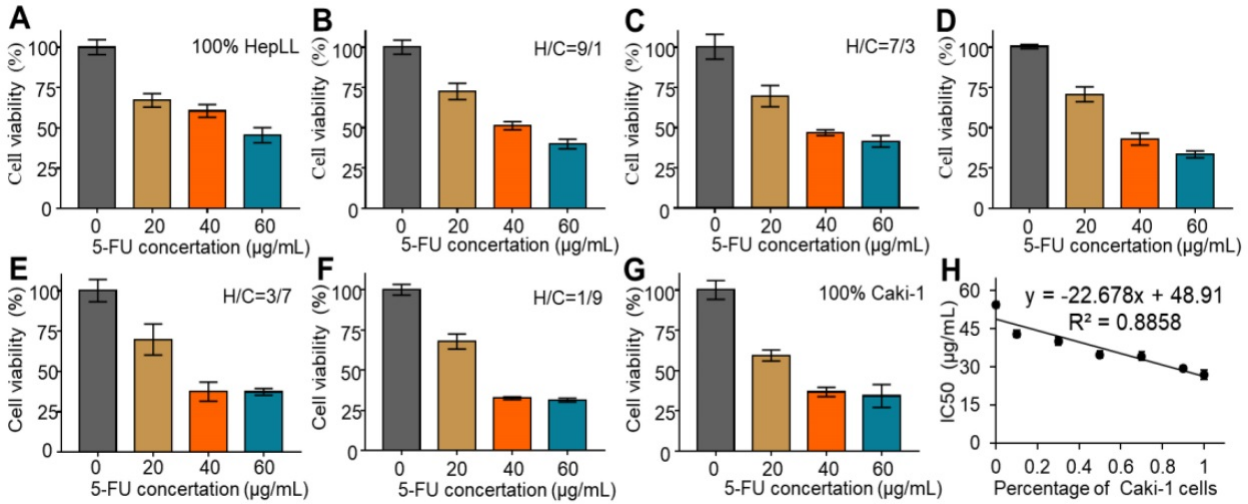
Cytotoxicity of 5-FU in the tumor progression model based on metastasis-on-a-chip. (A-G) Quantification of cytotoxicity of 5-FU for 100% HepLL, H/C=1/9, H/C=3/7, H/C=5/5, H/C=7/3, H/C=9/1 and 100% Caki-1, respectively, by a CCK-8 kit. (H) IC_50_ changes in different ratios of HepLL and Caki-1 cells. (Avg. ± SD, n = 3).

The metastatic tumor progression model was used to assess the efficacy of 5-FU in killing Caki-1 cells in liver-specific ECM. Cell viability was assessed over increasing concentrations of 5-FU (0, 20, 40, and 60 μg/mL) in the different growth stages of the metastatic tumor progression model. The CCK-8 assay results showed that the 5-FU treatment at 20, 40, and 60 μg/mL led to significant cell death after 24 hours, as opposed to the untreated group, regardless of cell ratios (Figure 5A-G). Furthermore, the IC_50_ was determined to be 54.56 ± 1.30 μg/mL, 43.01 ± 1.50 μg/mL, 39.99 ± 1.51 μg/ mL, 34.87 ± 1.48 μg/mL, 34.24 ± 1.63 μg/mL, 29.41 ± 0.87 μg/mL and 26.92 ± 1.94 μg/mL in 100% HepLL, H/C=9/1, H/C=7/3, H/C=5/5, H/C=3/7, H/C=1/9 and 100% Caki-1, respectively. The IC_50_ decreased with increasing Caki-1 percentage, indicative of the linear anticancer relationship between 5-FU concentration and the percentage of Caki-1 cells in the co-culture system (R^2^ = 0.89). The result showed that the more Caki-1 cells in the co-culture, the more cells were killed with the same dose of 5-FU, owing to HepLL cells being less sensitive to 5-FU (Figure 5H).

### Characterization of 5-FU-loaded PLGA-PEG NPs

**Figure 6:**
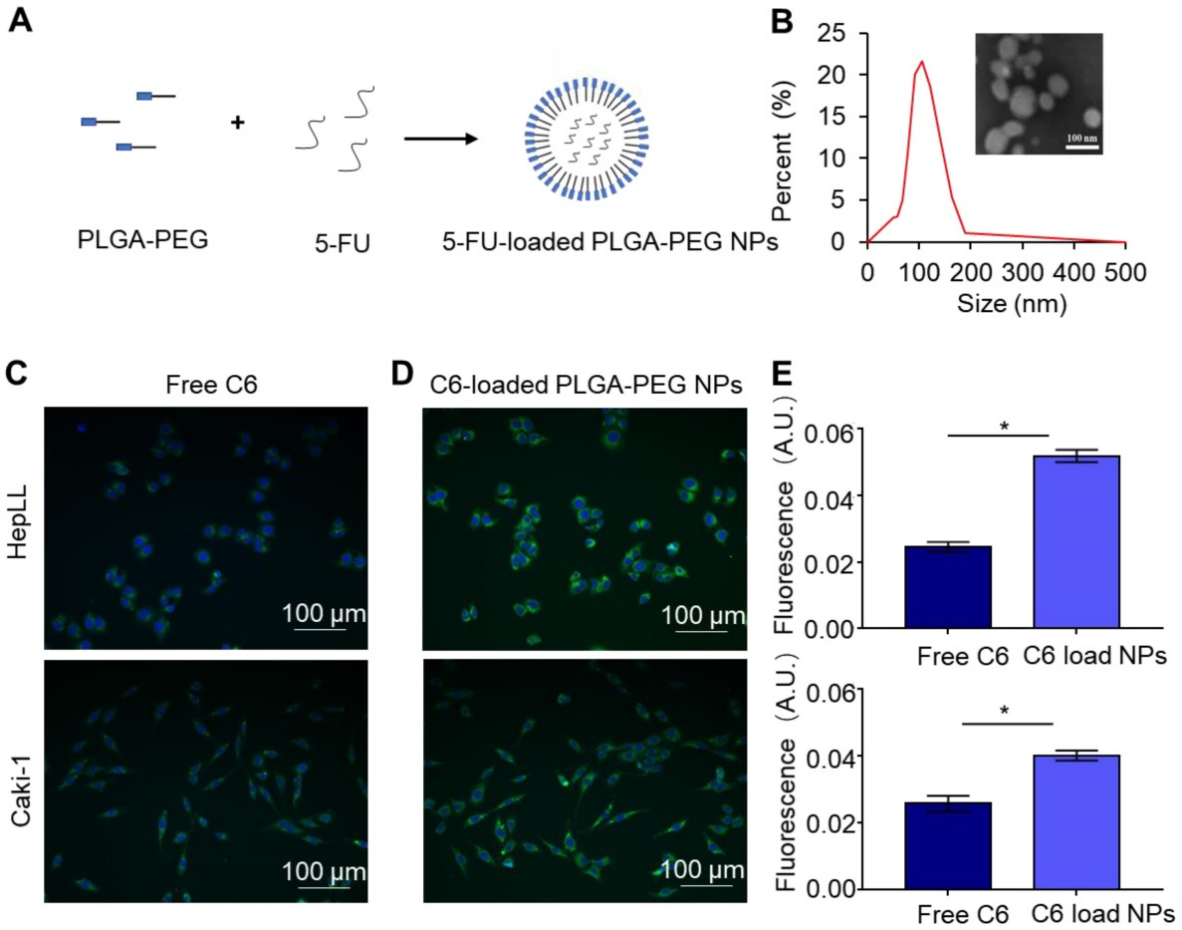
Characterization of 5-FU-loaded PLGA-PEG NPs and C6-loaded PLGA-PEG NPs co-cultured with HepLL and Caki-1 cells. (A) Schematic of NP-encapsulated 5-FU. (B) The size distribution and TEM of 5-FU-loaded PLGA-PEG NPs. Free C6 (C) and C6-loaded PLGA-PEG NPs (D) incubated with HepLL and Caki-1 cells for 24 h. (E) Comparison of fluorescence intensity of free C6 and C6-loaded PLGA-PEG NPs HepLL and Caki-1 cells. (Avg. ± SD, * stands for p < 0.05, n = 3).

The PLGA-PEG polymer, as a model delivery system, was evaluated using our tumor progression model to increase the anti-cancer effects of 5-FU (Figure 6A). The resultant 5-FU-loaded PLGA-PEG NPs were characterized by DLS and TEM. DLS analysis showed the size distribution of 5-FU-loaded PLGA-PEG NPs from 50.7 to 190 nm, with an average size of 106.58 nm (Figure 6B). TEM images validated the formation of spherical NPs using the NP synthesis method as previously described (Figure 6B). In addition, the 5-FU EE (%) within the PLGA-PEG NPs was determined to be 35.46 ± 0.87%.

### Delivery of C6 by PLGA-PEG NPs

The delivery efficacy of C6 by PLGA-PEG NPs was evaluated by co-culture of free C6 and C6-PLGA-PEG NPs with the HepLL and Caki-1 cells, respectively. Fluorescence microscopic images showed a significantly higher cellular uptake of C6-loaded NPs versus the free C6 (Figure 6C-D). When incubated with free C6 and NP-encapsulated C6, the mean cell fluorescence of free C6 were 0.024 ± 0.0011 and 0.025 ± 0.0017 for HepLL and Caki-1 cells, while the mean cell fluorescence of C6-PLGA-PEG NPs were 0.052 ± 0.0015 and 0.04 ± 0.0019, respectively. Both HepLL and Caki-1 cells had significantly higher normalized mean cell fluorescence (p < 0.05) when incubated with the C6-loaded NPs (Figure 6E), indicating that the NPs efficiently delivered C6 to the cells.

### Delivery of 5-FU by PLGA-PEG NPs in metastatic tumor progression model

**Figure 7:**
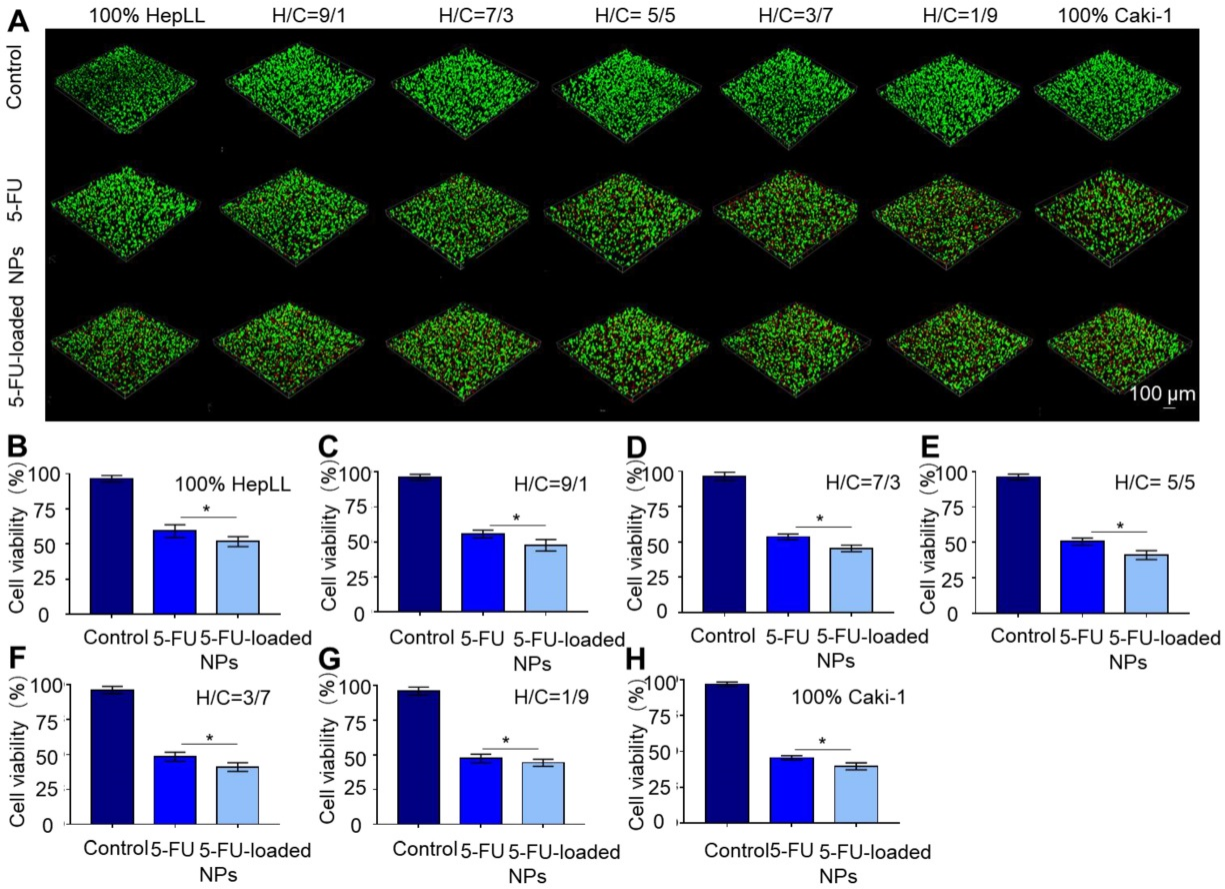
Evaluation of 5-FU-loaded PLGA-PEG NPs' toxicity using the metastatic tumor progression model. (A) Live/dead staining of HepLL and Caki-1 cells as well as the effects of 5-FU and 5-FU-loaded NPs on cell viability at 24 hours. (B-H) Cell viability of various ratios of HepLL to Caki-1 cells treated with 30 μg/mL of 5-FU or 5-FU-loaded NPs in the tumor progression model based on metastasis-on-a-chip at 24 hours. (Avg. ± SD, * stands for p < 0.05, n = 3).

The delivery of 5-FU by PLGA-PEG NPs to the metastatic tumor progression model was confirmed by live/dead staining. Cell viability was measured over increasing concentrations of 5-FU and 30 μg/mL was chosen as the working concentration based on the IC_50_ results. The overlaid confocal images cells following live/dead staining illustrated that 5-FU and 5-FU-loaded PLGA-PEG NP treatment at a concentration of 30 μg/mL resulted in significant (p < 0.05) cell death after 24 hours, as opposed to the untreated group (Figure 7A). The cell viability was 59.26 ± 4.57%, 55.52 ± 2.82%, 53.54 ± 2.11%, 50.53 ± 2.47%, 48.49 ± 3.19%, 47.40 ± 3.04% and 45.54 ± 1.50% after 24 hours of co-culturing with 30 μg/mL of 5-FU at an increasing ratio of Caki-1 cells. Meanwhile, the cell viability was 51.75 ± 3.56%, 47.68 ± 4.09%, 45.46 ± 2.32%, 41.09 ± 3.12%, 41.07 ± 3.11%, 44.27 ± 2.49% and 39.53 ± 2.50% for 100% HepLL, H/C=9/1, H/C=7/3, H/C=5/5, H/C=3/7, H/C=1/9 and 100% Caki-1 cells after 24 hours of co-culture with 30 μg/mL of 5-FU-loaded PLGA-PEG NPs, respectively. These results showed that 5-FU-loaded PLGA-PEG NPs were significantly more effective than free 5-FU in killing Caki-1 cells (p < 0.05) (Figure 7B-H).

## Discussion and conclusion

**Figure 1:**
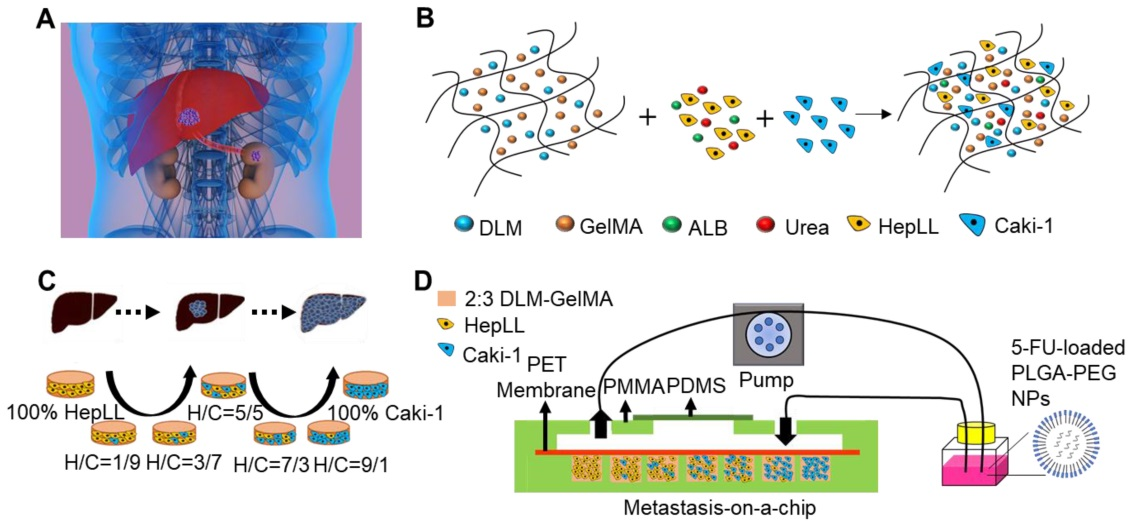
Schematic of the tumor progression model based on metastasis-on-a-chip. (A) Physiological representation of kidney cancer post-metastasis into the liver. (B) Diagram of the integration of co-culture by combining immortalized hepatocytes HepLL and kidney cancer Caki-1 cells into the ECM of 2:3 DLM/GelMA hydrogel. (C) Co-cultures of different ratios of HepLL to Caki-1 cells to mimic the progression of kidney cancer cells metastasized to the liver. (D) The efficacy of 5-FU, delivery of 5-FU though PLGA-PEG NPs, and dose optimization can be evaluated on the metastasis-on-a-chip platform.

Organ-specific ECM plays an important role in metastatic cancer; therefore, we simulated the biomimetic liver microenvironment in a tumor progression model based on metastasis-on-a-chip. In this study, we cultured Caki-1 cells in 3D biomimetic liver microenvironments (2:3 DLM/GelMA) to mimic the progression of metastatic kidney cancer in a metastasis-on-a-chip model (Figure 1 and S1). The decellularized scaffold retains collagen I, collagen IV, fibronectin, and laminin (Figure 2), which are essential for tumor growth and metastasis 36-38. As for biophysical cues, the Young's modulus obtained from the 2:3 DLM/GelMA (Figure 2H) is similar to that of a fibrotic liver (> 12 kPa) 39, and is within the liver stiffness range of liver cancer (approximately 5 to 60 kPa) 34, 35. In addition, a flow of fluid (2 μL/h) was implemented in the microfluidic system, not only providing the required shear stress to the cells, but also facilitating the exchange of oxygen and removal of metabolic waste. Through the construction of the organ-specific ECM, HepLL cells were able to continuously produce albumin and urea, mimicking the biological functions of liver. These results have suggested that DLM/GelMA-based biomimetic liver microtissues can mimic the organ-specific ECM for studying the progression of kidney cancer metastasized to the liver.

To create tumor progression model based on metastasis-on-a-chip, we co-cultured different ratios of HepLL and Caki-1 cells in the organ-specific ECM. Transduced HepLL cells expressing green fluorescent protein and transduced Caki-1 cells expressing red fluorescent protein were successfully co-cultured with different cell ratios (Figure 3B-D). In addition, we used live/dead staining to assess long-term cell viability. To avoid fluorescence overlap in transduced cells, we used non-transduced cells to better study cell viability and function. The cell viability was greater than 90% at day 14, and HepLL cells continuously produced high levels of ALB and urea (Figure 4). Thus, this metastatic tumor progression model was used for long-term cell co-culture at an increasing ratio from 1:9 to 9:1 in a liver-specific ECM, and partial functions of hepatocytes were maintained. Although metastasis-on-a-chip models were used to study the migration of cancer cells and offer a feasible platform for drug testing 25, 27, 40, the progression of post-metastasis tumor within an organ-specific ECM has not been studied. Therefore, we mimic the post-metastasis progression through co-cultures of various ratios of HepLL and Caki-1 cells in a liver-specific ECM.

The tumor progression model based on metastasis-on-a-chip can predict the therapeutic effects and evaluate the efficacy of anticancer drugs in inhibiting the growth of metastatic cancer cells. This metastatic tumor progression model showed the efficacy of 5-FU as well as a linear anticancer relationship between the concentration of 5-FU and the percentage of Caki-1 cells in the co-culture system (R^2^ = 0.89) (Figure 5). In addition, 5-FU-loaded PLGA-PEG NPs showed a stronger killing efficacy than free 5-FU at the same concentration (30 μg/mL) due to the delivery abilities of NPs (Figure 7), which is observed in a 2D model 33. Compared to conventional 2D cultures and 3D Transwell, this bionic tumor progression model based on metastasis-on-a-chip with organ-specific ECM can better represent *in vitro* systems to study the efficacy of naked and carrier loaded drugs. On the other hand, unlike animal models, the tumor progression model based on metastasis-on-a-chip is less expensive, less labor intensive, and is not hindered by differences between animal and human cell responses. Therefore, the tumor progression model based on metastasis-on-a-chip can be used for accurate evaluation and prediction of the effects of drug treatment and drug delivery in an organ-specific ECM.

Although the tumor progression model based on metastasis-on-a-chip is effective for drug evaluation, our study may suffer from the following limitations. First, we used the rat liver instead of the human liver in preparing ECM despite the disparity between Homo sapiens and Rodentia. Since DLM is commonly prepared by perfusing a series of detergents through the portal vein, it would be clinically challenging to collect whole human livers and to carry out the perfusion process for preparing human liver ECM 41. Nevertheless, it has been shown that rat liver ECM has significant similarities with human liver ECM since both contain key growth factors and major collagens, which are key components in composing of biological and biomechanical cues in the liver 41, 42. Second, it would be ideal to recapitulate the (pre)metastatic niche by using the liver ECM derived from a xenografted RCC model in our study. It has been previously reported that the primary tumor remodels the ECM in the metastatic sites distantly through soluble factors, exosomes and other microenvironmental cues for preparing the “soil” for “seeds”, *i.e.,* circulating tumor cells to reside and grow 43, 44. Since the (pre) metastatic niche clearly affects cell homing, colonization, adhesion, proliferation, and differentiation 45, it would be better to establish the (pre)metastatic ECM niche to a greater extent for studying metastasis and associated mechanisms. However, our research aims to establish a tumor progression model after the occurrence of metastasis for drug evaluation. As such, it is conceivable that the effect of using healthy liver ECM rather than (pre)metastatic ECM in our study would be less pronounced in causing bias for drug evaluation. Collectively, the use of healthy rat livers for preparing ECM, though not as ideal as human liver ECM or rat liver ECM derived from xenografted RCC, is more practical in preparing a post-metastasis tumor progression model on chip for preliminary drug evaluation.

In summary, we developed a tumor progression model based on metastasis-on-a-chip that cultures kidney cancer cells in a 3D biomimetic liver ECM to mimic the progression of kidney cancer cells for predicting therapeutic effects and assessing dosage response at different stages of tumor progression. The novel tumor progression model is unique in terms of *in vitro* organ-specific ECM working in conjunction with the microfluidic system for providing biological biophysical, and biomechanical cues. Considering the technical challenge to establish a RCC model metastasized to the liver in rats via intrasplenic or intrahepatic injection, the post-metastatic tumor progression model is more cost-effective and practical to screen for therapeutic compounds, evaluate drug delivery routes, and assess treatment potency in preliminary studies. In addition, this tumor progression model can be adapted and potentially used for developing personalized medicine for patients using patient-derived metastatic tumor tissues. Nevertheless, the translation of any key findings from tumor-on-a-chip platforms to clinical usage would require stringent validation through animal studies and clinical trials.
