# Supplementary material for: Metastasis-on-a-chip mimicking the progression of kidney cancer in the liver for predicting treatment efficacy
Source: Theranostics. 2020 Jan 1;10(1):300–11. doi: 10.7150/thno.38736 (PMC6929630; doi:10.7150/thno.38736)
Supplement: Supplementary file 1 — Supplementary figures and tables. [file thnov10p0300s1.pdf]

Supplementary Information

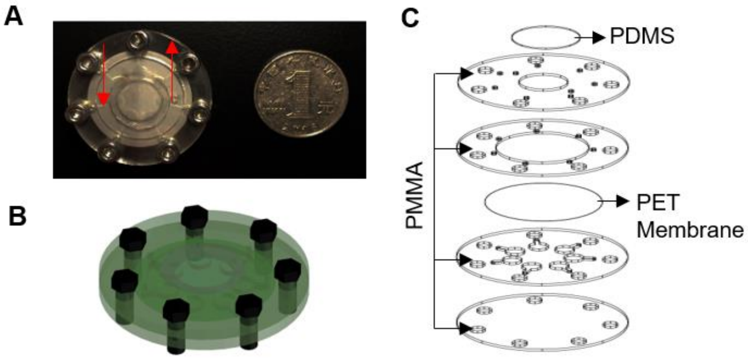

Figure S1. Design of the tumor progression model based on metastasis-on-a-chip. Photo (A) and 3D graphics (B) of the microfluidic device, for establishing a tumor progression model based on metastasis-on-a-chip (C).
